# Alcohol and Cancer: Epidemiology and Biological Mechanisms

**DOI:** 10.3390/nu13093173

**Published:** 2021-09-11

**Authors:** Harriet Rumgay, Neil Murphy, Pietro Ferrari, Isabelle Soerjomataram

**Affiliations:** 1 Cancer Surveillance Branch, International Agency for Research on Cancer, CEDEX 08, 69372 Lyon, France; SoerjomataramI@iarc.fr; 2 Nutrition and Metabolism Branch, International Agency for Research on Cancer, CEDEX 08, 69372 Lyon, France; murphyn@iarc.fr (N.M.); ferrarip@iarc.fr (P.F.)

**Keywords:** alcohol, acetaldehyde, oxidative stress, inflammation, one carbon metabolism, lipid metabolism, DNA damage, cancer, carcinogenesis

## Abstract

Approximately 4% of cancers worldwide are caused by alcohol consumption. Drinking alcohol increases the risk of several cancer types, including cancers of the upper aerodigestive tract, liver, colorectum, and breast. In this review, we summarise the epidemiological evidence on alcohol and cancer risk and the mechanistic evidence of alcohol-mediated carcinogenesis. There are several mechanistic pathways by which the consumption of alcohol, as ethanol, is known to cause cancer, though some are still not fully understood. Ethanol’s metabolite acetaldehyde can cause DNA damage and block DNA synthesis and repair, whilst both ethanol and acetaldehyde can disrupt DNA methylation. Ethanol can also induce inflammation and oxidative stress leading to lipid peroxidation and further DNA damage. One-carbon metabolism and folate levels are also impaired by ethanol. Other known mechanisms are discussed. Further understanding of the carcinogenic properties of alcohol and its metabolites will inform future research, but there is already a need for comprehensive alcohol control and cancer prevention strategies to reduce the burden of cancer attributable to alcohol.

## 1. Introduction

**Figure 1:**
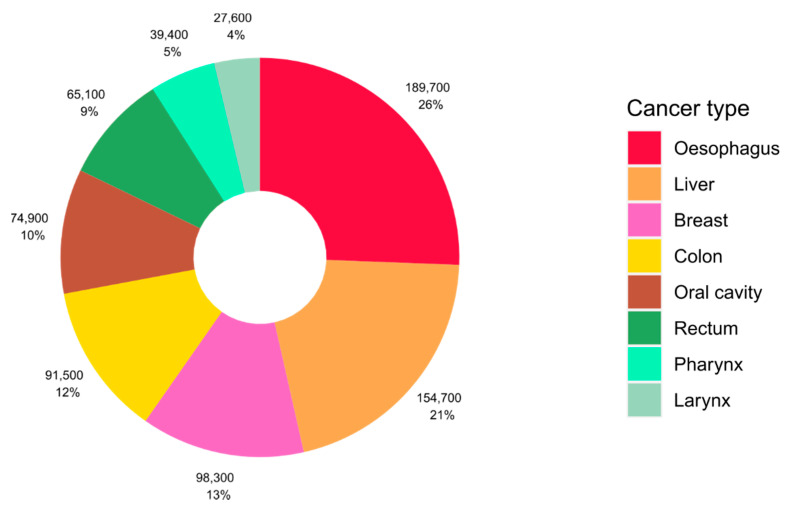
Global number and proportion of cancer cases attributable to alcohol consumption according to cancer type. Source of alcohol-attributable cases: Rumgay and colleagues [1].

Approximately 4% of cancers worldwide are caused by alcohol consumption, equating to more than 740,000 cases of cancer globally in 2020 [1]. The impact of alcohol consumption on cancer burden differs by cancer type, and cancers of the oesophagus, liver, and breast represent the most alcohol-attributable cases of cancer globally (Figure 1). Drinking alcohol even at lower levels of intake can increase the risk of cancer and we previously estimated that over 100,000 cases of cancer in 2020 were caused by light and moderate drinking of the equivalent of around one or two alcoholic drinks per day [1]. Despite this, there is low public awareness of the causal link between alcohol and cancer and alcohol use is growing in several regions of the world [2,3].

More than 30 years ago, in 1988, the International Agency for Research on Cancer (IARC) classified alcoholic beverages as a group 1 carcinogen, the most severe classification [4]. The IARC Monographs program aims to classify cancerous agents according to the strength of the available epidemiological and experimental evidence. Cancers of the oral cavity, pharynx, larynx, oesophagus, and liver were first classified as being causally related to the consumption of alcoholic beverages, and this was expanded to include cancers of the colorectum and female breast in the later monographs on alcoholic beverages in 2010 and 2012, with a positive association observed for cancer of the pancreas [5,6].

The World Cancer Research Fund (WCRF) also conducts classification of physical and dietary components and their potential cancerous effects as part of their Continuous Update Project. The WCRF base their conclusions on the quality of epidemiological evidence and carry out meta-analyses of the association with cancer risk. In the most recent report on Diet, Nutrition, Physical Activity and Cancer, WCRF concluded that there was strong evidence that alcohol consumption increased the risk of cancers of the mouth, pharynx and larynx, oesophagus (squamous cell carcinoma), liver, colorectum, and breast (postmenopausal), with a probable increased risk of stomach cancer and premenopausal breast cancer [7].

In addition to associations from epidemiological studies, multiple mechanistic pathways through which alcohol can cause cancer have been proposed. In this review, we aim to summarise the epidemiological evidence on alcohol and cancer risk and the mechanistic evidence of alcohol-driven carcinogenesis. We searched the PubMed and Cochrane databases for reviews, umbrella reviews, meta-analyses, and Mendelian randomisation studies on total alcohol use and cancer risk and mechanisms of alcohol-related carcinogenesis published up until June 2021. We also searched the WCRF’s Continuous Update Project reports for meta-analyses on alcohol consumption and cancer risk.

## 2. Alcohol and Cancer Risk

**Figure 2:**
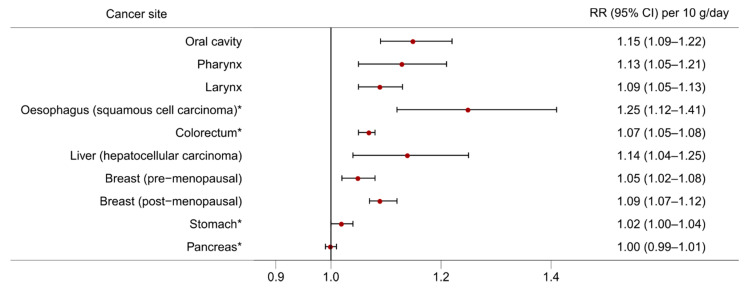
The dose-response relationship for the risk of cancer at different sites per 10 g/day increase in alcohol consumption. Source of relative risk estimates: WCRF Continuous Update Project [7]. RR = Relative risk; CI = Confidence interval. * Non-linear dose-response observed indicating threshold effect.

**Figure 3:**
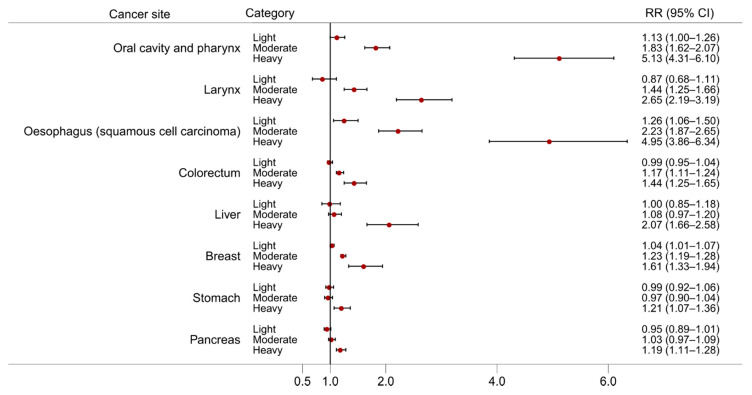
The dose-response relationship for the risk of cancer at different sites by three level of alcohol intake: light (up to 12.5 g/day), moderate (12.5 to 50 g/day), and heavy (more than 50 g/day). Source of relative risk estimates: Bagnardi and colleagues [8]. RR = Relative risk; CI = Confidence interval.

The effects of alcohol consumption on cancer risk have been studied for many decades and an association with alcohol has been observed for multiple cancer sites. Here, we discuss evidence from large meta-analyses of observational studies and emerging evidence from Mendelian randomisation studies. Figure 2 and Figure 3 present the dose-response relationships for the risk of cancer at several sites per 10 g/day increase in alcohol consumption from the meta-analyses carried out in the WCRF Continuous Update Project [7], and the risk of cancer at several sites according to three levels of alcohol intake [light (up to 12.5 g/day), moderate (12.5 to 50 g/day), and heavy (more than 50 g/day)] from a meta-analysis conducted by Bagnardi and colleagues [8], both with respect to the reference category of alcohol non-drinkers.

### 2.1. Oral Cavity Pharyngeal and Laryngeal Cancers

Drinking alcohol increases the risk of cancers of the upper aerodigestive tract. Consumption of 10 g alcohol per day was associated with a 15% increased risk of oral cavity cancer (RR 1.15 (95% CI 1.09–1.22)) in the most recent WCRF Continuous Update Project [7]. Pharyngeal cancer risk was also increased (RR 1.13 (95% CI 1.05–1.21) per 10 g alcohol per day) [7]. In Bagnardi and colleagues’ meta-analysis the RR of cancer of the oral cavity and pharynx was increased from 1.13 (95% CI 1.00–1.26) for current light drinking (up to 12.5 g alcohol per day) to 5.13 (95% CI 4.31–6.10) for heavy drinking (more than 50 g per day) [8]. Cancers of the larynx were also observed to have an increased RR (1.09 (95% CI 1.05–1.13) per 10 g alcohol per day) in the WCRF meta-analysis [7]. Bagnardi and colleagues found significant increases in laryngeal cancer risk only in moderate and heavy drinking, with RRs of 1.44 (95% CI 1.25–1.66) and 2.65 (95% CI 2.19–3.19), respectively [8].

### 2.2. Oesophageal Cancer

Drinking alcohol increases the risk of squamous cell carcinoma of the oesophagus which is the most common histological subtype of oesophageal cancer globally, and contributed the most cases of cancer in 2020 attributable to alcohol (189,700 cases) [1,9]. An excess risk of oesophageal squamous cell carcinoma was found in the WCRF Continuous Update Project (RR 1.25 [95% CI 1.12–1.41] per 10 g alcohol per day) [7], and in Bagnardi and colleagues’ meta-analysis, the pooled RR estimates for light and heavy drinking were 1.26 (95% CI 1.06–1.50) and 4.95 (95% CI 3.86–6.34), respectively [8]. There were differences in risk between geographic locations in both meta-analyses, with higher oesophageal squamous cell carcinoma risk among drinkers in studies conducted in Asia than those in North America or Europe. This observation possibly reflects the elevated risk of oesophageal squamous cell carcinoma among carriers of the ALDH2*2 polymorphism of the gene that codes the enzyme aldehyde dehydrogenase 2 (ALDH2) [10]. The ALDH2*2 variant allele is more common in Eastern Asian populations and confers nearly four times the risk of oesophageal cancer among drinkers compared with ALDH2*1 carriers [10]. For oesophageal adenocarcinoma, the second most common histological subtype of oesophageal cancer, no increased risk was observed in the WCRF meta-analysis (RR 1.00 (95% CI 0.98–1.02) per 10 g per day) but an inverse association was found for oesophageal adenocarcinoma and gastric cardia cancer among light drinkers in the meta-analysis by Bagnardi and colleagues (RR 0.86 (95% CI 0.76–0.98)) [7,8].

Cancers of the upper aerodigestive tract can also be characterised as having a more than multiplicative increased risk when alcohol and tobacco are consumed together. This synergistic effect has been observed in several studies; for example a pooled analysis of 11,200 head and neck cancer cases and 16,200 controls found a 14 times risk of head and neck cancers among those who drank at least three alcoholic drinks per day and smoked more than 20 cigarettes per day, compared with never drinkers who had never smoked [11]. For oesophageal squamous cell carcinoma, a cohort study in the Netherlands observed an eight times risk among current smokers who drank 15 g alcohol or more per day, compared with never smokers who consumed less than 5 g alcohol per day [12].

### 2.3. Colorectal Cancer

The meta-analysis conducted by WCRF found a 7% increased risk of colorectal cancer (RR 1.07 (95% CI 1.05–1.08)) per 10 g alcohol per day [7]. WCRF also found some evidence of a threshold effect around 20 g per day with a weaker association at lower intake levels [7]. The meta-analysis by Bagnardi and colleagues did not find an effect of alcohol on colorectal cancer risk among light drinkers, but the RR increased to 1.17 (95% CI 1.11–1.24) for moderate drinking, and 1.44 (95% CI 1.25–1.65) for heavy drinking [8]. Differences between subsites were minimal, with the risk of colon cancer (RR 1.07 95% CI 1.05–1.09) similar to rectal cancer (RR 1.08 95% CI 1.07–1.10) [7]. Alcohol might also increase the risk of precancerous lesions in the colon, with a meta-analysis reporting a 27% increased risk of colorectal adenoma (RR 1.27 (95% CI 1.17–1.37)) per 25 g alcohol per day [13].

### 2.4. Liver Cancer

The most common histological subtype of liver cancer is hepatocellular carcinoma (HCC) and around 154,700 cases of HCC in 2020 were attributable to alcohol consumption [1]. When restricted to HCC only, meta-analysis of WCRF sources resulted in a 14% increased risk of HCC (RR 1.14 (95% CI 1.04–1.25)) per 10 g alcohol per day [7]. However, a possible threshold effect was observed in the non-linear dose-response analysis by WCRF, where less than 45 g alcohol per day did not significantly increase the risk of liver cancer. This was similar to the findings of Bagnardi and colleagues where light or moderate drinking did not significantly increase liver cancer risk but risk among heavy drinkers doubled (RR 2.07 (95% CI 1.66–2.58)) [8].

### 2.5. Breast Cancer

Female breast cancer is the most commonly diagnosed cancer globally and contributed the third largest number of alcohol-attributable cases in 2020 (98,300 cases) [1,14]. The WCRF found a 7% increased risk of breast cancer per 10 g alcohol per day (95% CI 1.05–1.09) [7]. Whether there is a difference in breast cancer risk by menopausal status is unclear, as risk of postmenopausal breast cancer overlapped with that of premenopausal breast cancer in the WCRF meta-analysis (RR 1.09 (95% CI 1.07–1.12) versus RR 1.05 (95% CI 1.02–1.08), respectively, per 10 g alcohol per day). It does, however, seem that risk of breast cancer among drinkers might be specific to hormone receptor status; the WCRF meta-analysis of postmenopausal women observed an excess risk of oestrogen-receptor-positive and progesterone receptor-positive (ER^+^PR^+^) tumours (RR 1.06 (95% CI 1.03–1.09)) and ER^+^PR^–^ tumours (RR 1.12 (95% CI 1.01–1.24)) per 10 g alcohol per day, and no significant association was observed for ER^–^PR^–^ tumours (RR 1.02 95% CI 0.98–1.06) [7]. In a meta-analysis by Sun and colleagues, current drinkers had an increased risk of all hormone receptor status breast tumours compared with never drinkers, but RRs were higher for ER^+^PR^+^ tumours (RR 1.40 95% CI 1.30–1.51) and ER^+^PR^–^ tumours (RR 1.39 95% CI 1.12–1.71) than ER^–^PR^–^ tumours (RR 1.21 95% CI 1.02–1.43) [15].

### 2.6. Stomach Cancer

Alcohol consumption might increase the risk of stomach cancer. The linear dose-response meta-analysis by WCRF resulted in a non-significant RR of 1.02 (95% CI 1.00–1.04) per 10 g alcohol per day, but the non-linear dose-response analysis found an increase in stomach cancer risk for intakes over 45 g alcohol per day [7]. The meta-analysis by Bagnardi and colleagues observed a 21% increased risk in heavy drinking (RR 1.21 95% CI 1.07–1.36), and no significant increase in light or moderate drinking categories [8].

### 2.7. Pancreatic Cancer

The meta-analysis by WCRF did not find an increased risk of pancreatic cancer per 10 g alcohol per day (RR 1.00 (95% CI 0.99–1.01)) but there was a possible threshold effect of increased risk for intakes of around 60 g per day (RR 1.17 (95% CI 1.05–1.29)) [7]. This was a similar finding to the meta-analysis by Bagnardi and colleagues which found no increased risk at light or moderate drinking but a significant RR of 1.19 (95% 1.11–1.28) for heavy drinking [8].

### 2.8. Other Cancer Types

The association between alcohol drinking and risk of other cancer types has been studied but without sufficient evidence to be classified in the IARC monographs or WCRF Continuous Update Project. Positive associations have been reported in some meta-analyses; for example, a 3% increase in lung cancer risk was observed per 10 g alcohol per day in the WCRF meta-analysis based on 28 studies (RR 1.03 (95% CI 1.01–1.04)) after excluding studies which did not control for smoking [7]. A positive association with lung cancer was only found for heavy drinkers in Bagnardi and colleagues’ meta-analysis, but this was probably due to residual confounding from smoking because alcohol use did not increase the risk of lung cancer among non-smokers [8]. Little evidence of an association between alcohol consumption and gallbladder cancer was found in the WCRF Continuous Update Project, but Bagnardi and colleagues found an excess risk of gallbladder cancer among heavy drinkers (RR 2.64 (95% CI 1.62–4.30)). WCRF found an elevated risk of malignant melanoma per 10 g alcohol per day (RR 1.08 (95% CI 1.03–1.13)), but no effect on basal cell carcinoma (RR 1.04 (95% CI 0.99–1.10)) or squamous cell carcinoma (RR 1.03 (95% CI 0.97–1.09)) risk [7]. An increased risk of prostate cancer was observed for light and moderate drinking in Bagnardi and colleagues’ meta-analysis but not in the dose-response analysis of one drink per day by WCRF [7,8].

WCRF found an inverse association between alcohol consumption and kidney cancer risk (RR 0.92 (95% CI 0.86–0.97) per 10 g per day) [7]. However, this association was restricted to light and moderate drinking in Bagnardi and colleagues’ meta-analysis (RR 0.92 (95% CI 0.86–0.99) and 0.79 (95% CI 0.72–0.86), respectively) [8]. The same meta-analysis also found significant inverse associations for the risk of thyroid cancer, Hodgkin lymphoma and non-Hodgkin lymphoma [8].

### 2.9. Confirming the Causal Relation Reported in Observational Studies

Many observational studies have been conducted to identify and define the risks from drinking alcohol and cancer development. Some limitations in these studies have been identified, such as lack of sufficient adjustment of confounding factors, for example tobacco smoking and alcohol consumption are both common risk factors for oral cavity cancer. There are also concerns around reverse causality, with the reference categories of alcohol non-drinkers possibly including former drinkers who still have an elevated risk of cancer. There are other concerns over the accuracy of recording of alcohol exposure data where bias may be incorporated through non-participation of heavy drinkers in health studies, and under-reporting of alcohol consumption by the study subjects.

One method which might overcome some of the limitations in observational studies is Mendelian randomisation (MR), which uses genetic variants to explore the causal relationship between exposure and disease outcome. Assuming that analyses are conducted appropriately, due to the random distribution of these genetic variants at birth, MR studies should be less prone to conventional confounding and reverse causality.

For oral and oropharyngeal cancer, an MR study using genetic data on 6000 oral or oropharyngeal cancer cases and 6600 controls found a positive causal effect of alcohol consumption independent of smoking [16]. The authors concluded that previous estimates of the association between alcohol and oral and oropharyngeal cancer from observational studies may have been underestimated [16]. Another MR study on UK Biobank data found that drinking alcohol, especially above the UK’s low-risk guideline of up to 14 units per week, was causally related with head and neck cancers, but not breast cancer [17]. A further updated MR study using UK Biobank data did not find an association between alcohol exposure and cancer of any site, though they noted limitations of a lack of precision in their analyses due to low variance explained by the single nucleotide polymorphisms [18]. An MR analysis by Ong and colleagues found no significant increase in breast cancer risk per genetically predicted drink per day (odds ratio 1.00 (95% CI 0.93–1.08)) [19].

The future potential of MR studies is yet to be discovered but disclosing potential sources of biases and confounding in observational studies is necessary to obtain robust estimates of the causal relationship between alcohol consumption and cancer risk.

## 3. Mechanisms of Alcohol-Driven Carcinogenesis

**Figure 4:**
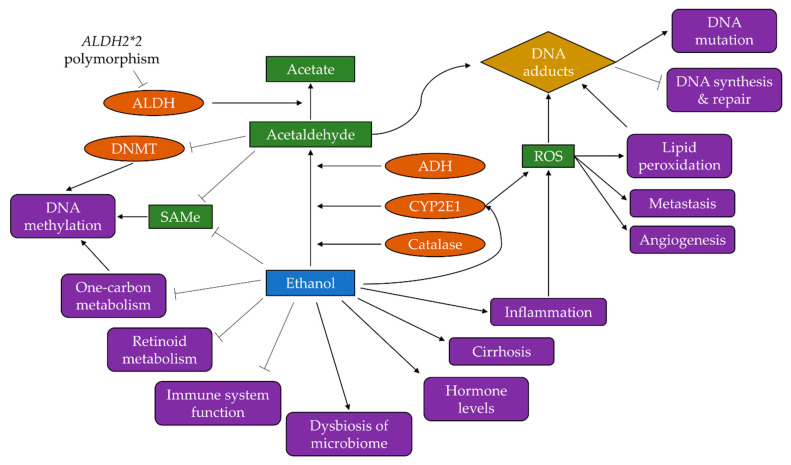
A simplification of the pathways by which alcohol, as ethanol, might drive carcinogenesis. The enzymes alcohol dehydrogenase (ADH), cytochrome P-450 2E1 (CYP2E1), and catalase metabolise ethanol to acetaldehyde; acetaldehyde dehydrogenase (ALDH) enzymes then metabolise acetaldehyde to acetate but common polymorphisms can reduce ALDH activity. Acetaldehyde forms DNA adducts causing mutations and blocking DNA synthesis and repair. Both ethanol and acetaldehyde can disrupt DNA methylation by inhibiting S-adenosyl-L-methionine (SAMe) synthesis and DNA methyltransferase (DNMT) activity, and ethanol can impair one-carbon metabolism. Cytochrome P-450 2E1 (CYP2E1) activity produces reactive oxygen species (ROS) leading to lipid peroxidation, metastasis, angiogenesis, and further formation of DNA adducts. Ethanol can also induce inflammation leading to production of ROS and their downstream effects. Retinoid metabolism and the normal function of the immune system are both impaired by ethanol, while ethanol may lead to increases in sex hormone levels, as well as dysbiosis of the microbiome and liver cirrhosis.

Following epidemiological evidence of the link between alcohol use and risk of cancer at multiple sites, several pathways have been investigated to explain the carcinogenic effects of alcohol. Here, we discuss the key mechanisms linking alcohol consumption to carcinogenesis, which are depicted in Figure 4.

### 3.1. Production of Acetaldehyde

Once consumed, alcohol is metabolised by enzymes including alcohol dehydrogenase (ADH), cytochrome P-450 2E1 (CYP2E1) and bacterial catalase, producing acetaldehyde [20]. Acetaldehyde is highly reactive towards DNA and has several carcinogenic and genotoxic properties.

As it is highly reactive towards DNA, acetaldehyde may bind to DNA to form DNA adducts which alter its physical shape and potentially block DNA synthesis and repair [21]. These DNA adducts are particularly genotoxic as they can induce DNA point mutations, double-strand breaks, sister chromatid exchanges, and structural changes to chromosomes [21,22]. The DNA adducts in question include N2-ethylidene-2′-deoxyguanosine, N2-ethyl-2′-deoxyguanosine, N2-propano-2′-deoxyguanosine (PdG), and N2-etheno-2′-deoxyguanosine [23]. The PdG adduct may form additional highly genotoxic structures such as DNA-protein cross-links and DNA interstrand cross-links which may confer carcinogenesis [24]. As well as DNA-protein cross-links, acetaldehyde may also bind to proteins directly causing structural and functional changes [21]; these proteins include glutathione, a protein involved in reducing oxidative stress caused by alcohol, and enzymes which contribute to DNA repair and methylation, among others.

Both acetaldehyde and ethanol can impact DNA methylation which may lead to changes in the expression of oncogenes and tumour-suppressor genes [21]. Acetaldehyde can inhibit the activity of DNA methyltransferase (DNMT) which is essential for normal DNA methylation; acetaldehyde can also reduce DNMT mRNA levels leading to less production of DNMT [25]. Acetaldehyde and ethanol may also inhibit the synthesis of S-adenosyl-L-methionine (SAMe) which is essential to DNA methylation [21].

Acetaldehyde is not the end-product of ethanol metabolism, however, as under normal conditions, acetaldehyde dehydrogenase (ALDH) enzymes convert acetaldehyde to acetate. The group of ALDH enzymes contains ALDH1A1, ALDH2, and ALDH1B1, with ALDH2 being responsible for the majority of acetaldehyde oxidation in the liver. A common polymorphism of this enzyme is the ALDH2*2 variant allele which dramatically reduces the activity of ALDH2 [10]. It is estimated that between 28% and 45% of East-Asian populations are carriers of the ALDH2*2 allele [10], while the proportion is considerably lower among Caucasians. In carriers of this polymorphism, acetaldehyde is not metabolised quickly enough, leading to an accumulation of acetaldehyde and thus the prolonged possibility to exert its described genotoxic effects. Evidence shows that alcohol drinkers who carry the ALDH2*2 variant allele have a substantially increased risk of cancers of the oesophagus and the upper aerodigestive tract [10], thus implicating the effects of acetaldehyde not only in the liver.

### 3.2. Induction of Oxidative Stress

Ethanol can also contribute to carcinogenesis through the induction of oxidative stress which is recognised as a key determinant of disease initiation [26]. Oxidative stress can be induced by activation of certain pathways which produce reactive oxygen species (ROS) such as superoxide anion and hydrogen peroxide. One pathway by which ethanol achieves this is through increased CYP2E1 activity which produces high quantities of ROS whilst oxidising ethanol to acetaldehyde [27]. Heavy alcohol use has been shown to increase *CYP2E1* expression in the oesophagus [27]. Other sources of ROS during ethanol metabolism include the mitochondrial respiratory chain and some cytosolic enzymes [28].

As ROS are highly reactive, their presence can lead to lipid peroxidation producing aldehydes which can bind to DNA forming etheno-DNA adducts [29,30]. These ethe-DNA adducts, namely 1,N6-ethenodeoxyadenosine and 3,N4-ethenodeoxycytidine, are highly mutagenic as they lead to mutations in several genes involved in key cell cycle regulation and tumour suppression [21]. Linhart and colleagues were able to demonstrate correlation between the amount of CYP2E1 and etheno-DNA adducts in cell, animal, and human tissue models, and highlighted their major importance in ethanol-mediated carcinogenesis in the liver, colorectum, and oesophagus, as well as other tissues [30].

Presence of ROS can also lead to changes in cell cycle behaviour. ROS can act as messengers in intracellular signalling pathways to activate the transcription factor nuclear factor κB (NF-κB). ROS can further promote cell proliferation and metastasis by interfering with mitogen-activated protein kinase signalling pathways and upregulating vascular endothelial growth factor (VEGF) and monocyte chemotactic protein-1 (MCP-1) which can stimulate angiogenesis [31]. In HCC tissue samples from alcohol drinkers, ROS accumulation and increased synthesis of VEGF, MCP-1 and NF-κB were observed, indicating alcohol-driven promotion and progression of HCC [32].

### 3.3. Increased Inflammation

Inflammation is a key pathway to cancer progression at several sites and is enhanced by alcohol use. Chronic alcohol consumption can recruit specific white blood cells (monocytes and macrophages) to the tumour microenvironment. These white blood cells produce pro-inflammatory cytokines, such as tumour necrosis factor α (TNF-α) and the interleukins IL-1, IL-6, and IL-8 [31,33], which activate oxidant-generating enzymes leading to downstream formation of ROS [30]. NF-κB is also activated by these cytokines, stimulating further ROS-producing enzymes.

In addition to its involvement in downstream ROS-producing pathways, it is hypothesised that IL-8 contributes to further accumulation of white blood cells (neutrophils, specifically) in the liver leading to acute inflammation. Elevated IL-8 levels have been found in patients with acute liver injury such as alcoholic hepatitis [34]. Additionally, the cytokine IL-6 stimulates production of the anti-apoptotic protein Mcl-1, thus avoiding cell death and exposing the cell to further DNA damage [35].

### 3.4. Disruption to One-Carbon Metabolism and Folate Absorption

There is mounting evidence that alcohol can negatively affect one-carbon metabolism which is essential for DNA methylation and DNA synthesis [25]. Ethanol and acetaldehyde can reduce the activity of enzymes involved in one-carbon metabolism that regulate DNA methylation, namely methionine synthase, methionine adenosyl transferase and DNMT, thus dysregulating epigenetic patterns and resulting in DNA hypomethylation [20].

Lipotropic nutrients such as folate are key sources of the methyl groups necessary for DNA methylation and influence the availability of SAMe, which is also essential to DNA methylation [25]. Alcohol intake may deplete folate levels, or indeed be a cause of folate and B vitamin deficiency if alcohol constitutes the majority of calories consumed, as observed in malnourished alcoholics [21,26]. Folate deficiency affects the availability of nucleotides needed for DNA synthesis leading to accumulation of deoxyuridine monophosphate which is incorporated into new DNA molecules causing double-strand breaks and chromosomal damage [25]. Interestingly, there is evidence that higher folate intake among alcohol drinkers may attenuate the increased risk of liver cancer mortality compared with those with low folate intake [36]. This attenuation was also observed for risk of postmenopausal breast cancer among women who drink alcohol and have higher folate levels [37]. The effect of alcohol on one-carbon metabolism and folate might also be important in colorectal cancer development [20].

### 3.5. Altered Retinoid Metabolism

Retinoids are important regulators against carcinogenesis as they can induce cell growth, cell differentiation, and apoptosis [31]. Alcohol can alter retinoid metabolism by inhibiting the oxidation of vitamin A to retinoic acid [21]. Alcohol increases CYP2E1 activity (Section 3.2) which also functions to metabolise retinoic acid resulting in the production of toxic metabolites [21]. This increased toxicity of retinoids may explain the observation of excess lung cancer risk in smokers who took β-carotene supplements and consumed 11 g or more of ethanol per day in the α-tocopherol, β-carotene cancer prevention study (ATBC trial) study [21].

Chronic alcohol consumption has been linked with decreased levels of retinoids in the liver [21], and low levels of retinol in the blood have been linked with higher risk of head and neck cancers [31]. Retinoids may also play a role in other signalling pathways implicated in cancer development, such as oestrogen and breast cancer [31].

### 3.6. Changes to Oestrogen Regulation

Alcohol might interfere with oestrogen pathways by increasing hormone levels and enhancing the activity of ERs, important in breast carcinogenesis [38]. Sex hormone levels may be increased by alcohol through oxidative stress and through inhibition of the steroid degradation enzymes sulfotransferase and 2-hydroxylase [39]. Heavy use of alcohol has also been linked with increased circulating levels of oestrone and oestradiol as well as dehydroepiandrosterone sulphate (DHEAS) [39]. DHEAS is metabolised to oestrogen by aromatase, the activity of which is also increased in chronic alcohol consumers [40]. A large cohort study found DHEAS levels 25% higher among women consuming at least 20 g alcohol per day compared with non-drinkers [41]. However, some of the associations among alcohol drinking premenopausal women were limited to those taking oral contraceptives [40]. Despite limited evidence of mediation of the association between alcohol and breast cancer by individual sex hormones, a case-control study nested within EPIC found that a hormonal signature reflecting lower levels of sex-hormone binding globulin and higher levels of sex hormones mediated 24% of the association, suggesting that an interplay of hormones may contribute to alcohol-mediated breast cancer development [42].

ERs are important transcription factors within cells and may provide the main pathway by which alcohol promotes breast tumour growth [40]. Elevated concentrations of oestrogen due to alcohol use may lead to increased transcriptional activity of ER (up to 15 times higher than normal activity), resulting in proliferation of ER^+^ cells [39].

### 3.7. Reduced Function of the Immune System

Alcohol has multiple negative effects on the host immune system. Firstly, alcohol can disrupt the production of proteins such as perforin and granzymes A and B, which are necessary for natural killer (NK) cells to function in targeting and destroying potentially cancerous cells [33]. Alcohol can block NK cells from being released from the bone marrow [31]. Alcohol can also activate NKT cells which are associated with liver injury and hepatocyte apoptosis [33]. Additionally, alcohol may suppress T cell immune responses therefore decreasing the anti-tumour regulation of the immune system.

With the immune system being compromised, alcohol consumption can exacerbate damage from viral infections such as hepatitis C virus, which is common among chronic alcoholic liver disease patients [43]. In addition, heavy episodic alcohol use might reduce the immune system’s defence against infection by disrupting the production of pro-inflammatory cytokines and increasing the expression of anti-inflammatory cytokines [33]. This is contrary to the increased expression of pro-inflammatory cytokines due to chronic alcohol exposure as discussed with other evidence on alcohol-induced inflammation (Section 3.3).

### 3.8. Dysbiosis of the Microbiome

Microbiota in the oral cavity metabolise ethanol to acetaldehyde by the enzyme catalase. However, these bacteria have limited capacity to break acetaldehyde down further into its non-harmful compound acetate, thus the oral epithelia are further exposed to acetaldehyde [21,44]. Acetaldehyde concentrations in the saliva of drinkers are between 10 and 100 times higher than in the blood; this is further doubled in smokers who drink alcohol as tobacco smoke contains high levels of acetaldehyde [21].

Increased ethanol consumption can induce microbial dysbiosis and bacterial overgrowth in the intestine [20]. This heightened bacterial presence may compromise the intestinal barrier resulting in ”gut leakiness” where the permeability of the intestinal lumen is high enough such that bacterial products including lipopolysaccharides and peptidoglycan move from the intestine into the blood [20,45]. Once in the blood these bacterial products easily reach the liver where a variety of cells are activated (endothelial cells, liver macrophages, stellate cells and hepatocytes) producing a chronic inflammatory environment [33], which may confer an increased risk of liver cancer [46].

### 3.9. Liver Cirrhosis

Liver cirrhosis is a well-recognised pathway to hepatocellular carcinoma development in heavy alcohol users and manifests as pre-neoplastic lesions in the liver [47]. Chronic alcohol exposure is associated with reduced expression of the cytokine interferon-γ which is an inhibitor of liver fibrosis [33]. Furthermore, ROS (Section 3.2) may trigger the production of pro-fibrotic cytokines and collagen in liver cells [28].

### 3.10. Activation of Other Carcinogens

There is further hypothesis that alcohol consumption might activate the pathways of other carcinogenic agents; this could occur through the alcohol-induced activity of CYP2E1 which may metabolise pro-carcinogens in tobacco smoke and industrial chemicals [21]. It is also possible that ethanol might aid these carcinogens to penetrate cells, especially those of the mucosa of the upper aerodigestive tract [21,48], where tobacco and alcohol have a synergistic effect on the risk of cancer [11,12].

## 4. Conclusions

Alcohol and its metabolite acetaldehyde can drive cancer development through several pathways. Many of these pathways are interlinked and show the complexity and breadth of alcohol’s harmful potential. For example, inflammation can result in oxidative stress, but inflammation is a reaction by the immune system which is itself compromised by alcohol use. Furthermore, DNA damage can occur through exposure to acetaldehyde and ROS which are both produced through CYP2E1 activity, with acetaldehyde also a product of ADH activity. Other potential pathways have been proposed including the dysregulation of carnitine metabolism [49]. We have only covered carcinogenesis in this review, but alcohol likely alters, through these pathways and others, other functions in the body which render it more susceptible to other diseases and injuries, as discussed in other articles in this Special Issue.

Alcohol consumption is a well-established risk factor for cancer and has been linked to cancers of the oral cavity and pharynx, oesophagus, liver, colorectum and breast. While studies have provided evidence on alcohol’s carcinogenic potential, further understanding of alcohol’s pathways to cancer development will inform the direction of future research. This information is useful to corroborate existing evidence, develop chemoprevention strategies, and could improve cancer therapy, but there is already a wealth of evidence to support the need for further alcohol control and cancer prevention efforts. We have discussed evidence on mechanistic and epidemiological research in the field, and this information must be used to decrease the burden of cancers, as well as other diseases and injuries, attributable to alcohol.
